# Neuropsychiatric Symptoms in Parkinson’s Disease Dementia Are More Similar to Alzheimer’s Disease than Dementia with Lewy Bodies: A Case-Control Study

**DOI:** 10.1371/journal.pone.0153989

**Published:** 2016-04-21

**Authors:** Pai-Yi Chiu, Chun-Tang Tsai, Ping-Kun Chen, Whe-Jen Chen, Te-Jen Lai

**Affiliations:** 1 Institute of Medicine, Chung Shan Medical University, Taichung, Taiwan; 2 Department of Neurology, Show-Chwan Memorial Hospital, Changhua, Taiwan; 3 Department of Guidance and Counseling, National Changhua University of Education, Changhua, Taiwan; 4 Department of Neurology, Lin-Shin Hospital, Taichung, Taiwan; 5 Department of Psychiatry, Chung Shan Medical University Hospital, Taichung, Taiwan; University Of São Paulo, BRAZIL

## Abstract

**Background and purpose:**

Previous studies on the clinical and pathological manifestations of Parkinson’s disease dementia (PDD) have reported findings more similar to dementia with Lewy bodies (DLB) than to Alzheimer’s disease (AD). The aim of this study was to investigate the neuropsychiatric symptoms of PDD compared to DLB and AD.

**Methods:**

We conducted a retrospective case-control study on 125 newly diagnosed consecutive PDD patients and age- and dementia stage-matched controls with either DLB (N = 250) or AD (N = 500) who visited the same hospital over the same period. For each case and control, neuropsychiatric symptoms were assessed using the Neuropsychiatric Inventory (NPI).

**Results:**

Overall, 513 (58.6%) patients were female and 362 (41.4%) were male. Comparisons of clinical data revealed that the PDD group, similar to the AD group, had a lower NPI total score, NPI caregiver burden score, and rate of antipsychotic use (all *p* < 0.001) than the DLB group. One or more psychiatric symptoms were reported in 95.2% of the PDD, 99.2% of the DLB, and 96.8% of the AD patients. The PDD group had lower subscores in the items of delusions, hallucinations, agitation, anxiety, irritation, aberrant motor behavior compared to the DLB group. Severe neuropsychiatric symptoms among all dementia patients were associated with younger age, more advanced stage, and a diagnosis of DLB.

**Conclusion:**

Neuropsychiatric symptoms in PDD were more like those in AD than in DLB. Severe neuropsychiatric symptoms in degenerative dementia were associated with younger age, more advanced stage of dementia, and a diagnosis of DLB.

## Introduction

Parkinson’s disease (PD) is the second most common neurodegenerative disorder worldwide, affecting approximately 0.4 to 1 percent among persons 60 to 79 years of age, rising to 1.9 percent among persons 80 years of age and older [1]. The average prevalence of dementia in PD is about 30–40%, with an incidence 4–6 times higher than that in the general age-appropriate population [2–4]. An 8-year prospective study has reported that nearly 80% of patients with PD progress to dementia after long-term follow-up [5]. The clinical diagnosis of dementia due to Parkinson’s disease (Parkinson’s disease dementia; PDD) is defined as dementia that occurs in the context of well-established Parkinson disease [3, 6]. Compared to dementia with Lewy bodies (DLB), the incident rate of PDD is substantially lower [7]. Besides, pathological confirmation of the accuracy of clinical diagnosis is also lower in PDD than in DLB [7]. McKeith et al. reported in the third consensus criteria for the diagnosis and management of DLB that other than age at onset, temporal course, and possibly response to levodopa, no major differences were found between PDD and DLB in clinical, neuropsychiatric, and pathological profiles [6]. Therefore, to differentiate PDD and DLB, the “one-year rule” has become the most commonly used operational tool for both clinical and research purposes, and the debate on whether PDD and DLB are the same disease entity continues [8–10]. Although most previous studies have demonstrated difficulties in differentiating PDD and DLB according to the manifestations of neuropsychiatric symptoms [6], the task force group of the Movement Disorder Society (MDS) proposed that patients with PDD who appear to have less frequent or less severe psychiatric symptoms than patients with DLB may simply reflect disparity in overall dementia severity [3]. Previous studies have included a relatively small number of cases [11–15], and only a few studies with large sample size but without matching age or disease severity have compared neuropsychiatric symptoms among patients with PDD, DLB, and Alzheimer’s disease (AD) [16–19]. Therefore, the first aim of this study was to investigate the similarities and differences of neuropsychiatric symptoms among PDD, DLB, and AD. The second was to clarify whether disease severity is contributing to the less frequent or less severe neuropsychiatric symptoms of patients with PDD compare to DLB. To achieve the goal, we enrolled a relatively large sample of patients with PDD compared to those in patients with DLB and AD, using a case-control study matched by age and disease severity according to the Clinical Dementia Rating (CDR) scale.

## Methods

### Participants

We conducted this retrospective case-control study on newly diagnosed consecutive PDD patients and age- (±3 years) and dementia stage- (same CDR or CDR-SB±1) matched controls with DLB or AD who visited the hospital over the same period at sample size ratios of 1: 2: 4, respectively, using a register-based database of all patients who visited the hospital’s dementia clinic from July 1, 2004 to June 30, 2013. The demographic data included age, onset age, gender, education, disease duration, disease severity, use of antipsychotics and use of antidepressants at the time of entry. The diagnosis of dementia was made according to the criteria for primary degenerative dementia in the fourth edition of the Diagnostic and Statistical Manual of Mental Disorders (DSM-IV); The PDD patients were diagnosed according to the clinical criteria for probable PDD developed by the MDS in 2007 [3]. The diagnosis of DLB was made according to the revised consensus criteria for probable or possible DLB developed by the third report of the DLB consortium (McKeith, 2005) [6]. The AD patients were diagnosed according to the criteria for probable and possible AD developed by the National Institute of Neurologic and Communicative Disorders and Stroke and the Alzheimer’s Disease and Related Disorders Association (NINCDS-ADRDA) [20].

### Assessment of neuropsychiatric symptoms

All patients and their main caregivers were interviewed by a trained neuropsychologist for assessment of the 12-item Neuropsychiatric Inventory (NPI) on the basis of observations within the past month. The NPI is a validated, standardized and widely used instrument that was developed specifically for neuropsychiatric symptoms of dementia [21, 22]. The 12-item NPI includes delusions, hallucinations, agitation, depression, anxiety, euphoria, apathy, disinhibition, irritation, aberrant motor behavior, night behavior, and eating/appetite behavior. All of the items were rated on symptom frequency from 1 (occasionally) to 4 (very frequently), on symptom severity from 1 (mild) to 3 (severe), and on caregiver burden from 0 (none) to 5 (extremely) [22].

### Assessment of disease severity, daily function, and cognitive function

The global severity of dementia was assessed according to the CDR scale and sum of boxes of CDR (CDR-SB). Daily function was assessed using the Instrumental Activities of Daily Living (IADL) Scale. Cognitive function was assessed using the Mini-mental State Examination (MMSE) and the Cognitive Abilities Screening Instrument (CASI) [23]. Cognitive tests for all patients were performed by a trained neuropsychologist. Diagnoses of dementia and subtypes of dementia were made by consensus in a meeting including two neurologists specializing in dementia (Chiu PY and Chen PK), one geriatric psychiatrist (Lai TJ), and one neuropsychologist (Tsai CT). The Hoehn and Yahr Scale of each patient with PDD was assessed by one of the neurologists (Chiu PY or Chen PK). All patients received at least cerebral CT or cerebral MRI and also blood screening tests for dementia.

### Data analysis

The Chinese version of SPSS 19.0 for Windows (IBM, SPSS Inc., Chicago) was used for statistical analyses. Comparisons among the PDD, DLB, and AD groups for demographic data, CDR-SB, IADL, CASI, MMSE, the composite score (frequency x severity) of the NPI, and the caregiver burden score of the NPI were analyzed using one-way ANOVA with either Bonferroni or Dunnett T3 post hoc analysis according to the homogeneity of variance. Gender, CDR, and the use of antipsychotics and antidepressants were analyzed with the chi-square test. Multiple logistic regression analysis was used to compare the associations of all patients with severe neuropsychiatric symptoms, diagnosis, CDR, age, gender, education, IADL, and MMSE among severe NPI and mild NPI groups. The cutoff point of NPI score between the mild and severe groups was defined as the median of the NPI score of all patients. A p value of less than 0.05 was considered to be statistically significant.

### Ethical consideration

The participants were selected from a register-based database of a regional hospital’s dementia clinic. The study design was retrospective and the data were analyzed anonymously. The Committee for Medical Research Ethics of Lin-Shin Hospital reviewed the project, and the Data Inspectorate approved the study.

## Results

A total of 875 patients were analyzed, including 125 (14.3%) with PDD, 250 (28.6%) with DLB, and 500 (57.1%) with AD. Among all patients, 513 (58.6%) were female and 362 (41.4%) were male. Table 1 compares the demographic data of the three dementia groups. The mean age of our PDD patients was relatively old (95.2% > 65 years old), and nearly half (46.4%) of the PDD patients had a short duration of parkinsonism (2–3 years). Comparisons of clinical and neuropsychiatric data revealed that the PDD group, similar to the AD group, had a lower rate of male gender, NPI total score, NPI caregiver burden score, and rate of currently using antipsychotics (all *p* < 0.001) than the DLB group.

**Table 1:** Comparison of demographic data among the PDD, DLB and AD groups.

Features	PDD	DLB	AD	f/χ2	*p*	Post-hoc/paired comparison
N	125	250	500			
Age, year (SD, range)	77.0 (7.0, 51–94)	78.2 (6.6, 51–91)	78.5 (8.0, 51–97)	1.98	NS	NA
CDR 0.5/1/2/3, N	25/52/39/9	45/100/87/18	102/210/159/29	1.86	NS	NA
H&Y stage 0/1/2/3/4/5, N	0/0/27/44/27/23	28/18/51/71/49/33				NA
Female, N (%)	72 (57.6)	116 (46.4)	325 (65.0)	**23.84**	**< 0.001**	PDD = AD > DLB
Education, year (SD, range)	6.0 (5.0, 0–16)	5.7 (4.8, 0–18)	5.1 (4.6, 0–18)	0.95	NS	PDD = DLB = AD
Onset age, year (SD, range)	74.7 (7.1, 54–94)	75.3 (8.3, 50–89)	75.3 (8.7, 48–95)	0.27	NS	PDD = DLB = AD
IADL (SD, range)	4.2 (2.6, 0–8)	4.1 (2.6, 0–8)	4.0 (2.6, 0–8)	0.21	NS	PDD = DLB = AD
CDR-SB (SD, range)	8.1 (4.0, 1.5–17)	8.5 (3.8, 1.5–17)	8.0 (3.5, 1.5–17)	1.70	NS	PDD = DLB = AD
MMSE (SD, range)	16.7 (7.4, 0–28)	15.1 (7.0, 0–28)	15.3 (7.1, 0–27)	2.23	NS	PDD = DLB = AD
CASI (SD, range)	52.6 (24.7, 0–90)	47.9 (23.4, 0–90)	49.4 (23.6, 0–89)	1.63	NS	PDD = DLB = AD
NPI-sum (SD, range)	21.3 (17.3, 0–85)	30.1 (18.9, 0–126)	23.0 (17.6, 0–99)	**15.19**	**< 0.001**	PDD = AD < DLB
NPI-burden (SD, range)	9.8 (8.0, 0–36)	13.7 (8.1, 0–44)	11.3 (8.1, 0–40)	**11.67**	**< 0.001**	PDD = AD < DLB
CHEIs, N (%)	39 (31.2)	97 (38.8)	158 (31.6)	4.25	NS	PDD = DLB = AD
Antipsychotics, N (%)	9 (7.2)	52 (20.8)	36 (7.2)	**33.51**	NS	PDD = AD < DLB
Antidepressants, N (%)	5 (4.0)	15 (6.0)	14 (2.8)	4.58	**< 0.001**	PDD = DLB = AD

PDD: Parkinson’s disease dementia; DLB: Dementia with Lewy bodies; AD: Alzheimer’s disease; NS: Non-significance; NA: Not applicable.

CDR: Clinical Dementia Rating Scale; H&Y stage: Hoehn and Yahr stage; IADL: Instrumental Activities of Daily Living; MMSE: Mini-Mental State Examination; CASI: Cognitive Abilities Screening Instrument; NPI: Neuropsychiatric Inventory; NPI-burden: Caregiver burden score in NPI. CHEIs: Current using Cholinesterase inhibitors (CHEIs); Antipsychotics: Current using antipsychotics; Antidepressants: Current using antidepressants.

One or more psychiatric symptoms were reported in 95.2% of the PDD, 99.2% of the DLB, and 96.8% of the AD patients. Table 2 shows comparisons of the subscores of the 12 items in the NPI among the three groups. After adjusting for gender and the use of antipsychotics, the PDD group had lower subscores in the items of delusions, hallucinations, agitation, anxiety, irritation, and aberrant motor behavior than the DLB group. The AD group had a similar neuropsychiatric profile to the PDD group and had lower subscores in the items of delusions, hallucinations, anxiety, aberrant motor behavior, sleep, and eat/appetite than the DLB group.

**Table 2:** Comparison of composite score (severity x frequency) of each item in the NPI among the PDD, DLB and AD groups adjusted for gender and use of antipsychotics.

	Mean (SD)			PDD vs. DLB		PDD vs. AD		DLB vs. AD	
Items	PDD	DLB	AD	OR	*p*	OR	*p*	OR	*p*
Delusion	1.7 (2.8)	2.8 (3.6)	2.0 (3.1)	**0.92**	**0.03**	0.97	NS	**1.06**	**0.013**
Hallucination	1.4 (2.8)	2.6 (3.5)	1.2 (2.5)	**0.89**	**0.004**	1.04	NS	**1.17**	**< 0.001**
Agitation	1.2 (2.4)	2.0 (2.9)	1.8 (2.8)	**0.91**	**0.048**	0.92	NS	1.01	NS
Depression	2.6 (3.6)	3.1 (3.2)	2.8 (3.1)	0.96	NS	0.99	NS	1.05	NS
Anxiety	2.3 (3.1)	2.8 (3.4)	2.2 (2.9)	**0.92**	**0.017**	1.01	NS	**1.10**	**< 0.001**
Euphoria	0.1 (0.6)	0.4 (1.9)	0.2 (1.0)	0.80	NS	0.87	NS	1.12	NS
Apathy	3.7 (4.1)	3.8 (3.9)	3.1 (3.5)	1.00	NS	1.04	NS	1.04	NS
Disinhibition	0.4 (1.6)	0.7 (1.8)	0.8 (2.2)	0.89	NS	0.89	NS	0.97	NS
Irritation	1.1 (2.2)	2.0 (3.1)	1.8 (2.8)	**0.89**	**0.011**	0.89	NS	1.02	NS
Aberrant motor behavior	1.0 (2.7)	2.2 (3.6)	1.5 (3.0)	**0.89**	**0.006**	0.93	NS	**1.05**	**0.048**
Sleep	4.3 (3.8)	5.4 (3.9)	4.2 (3.8)	0.94	NS	1.01	NS	**1.08**	**< 0.001**
Eat/Appetite	1.6 (3.0)	2.2 (3.5)	1.5 (2.8)	0.94	NS	1.02	NS	**1.09**	**0.001**

PDD: Parkinson’s disease dementia; DLB: Dementia with Lewy bodies; AD: Alzheimer’s disease; OR: Odds ratio; NS: Non-significance.

**Fig 1:**
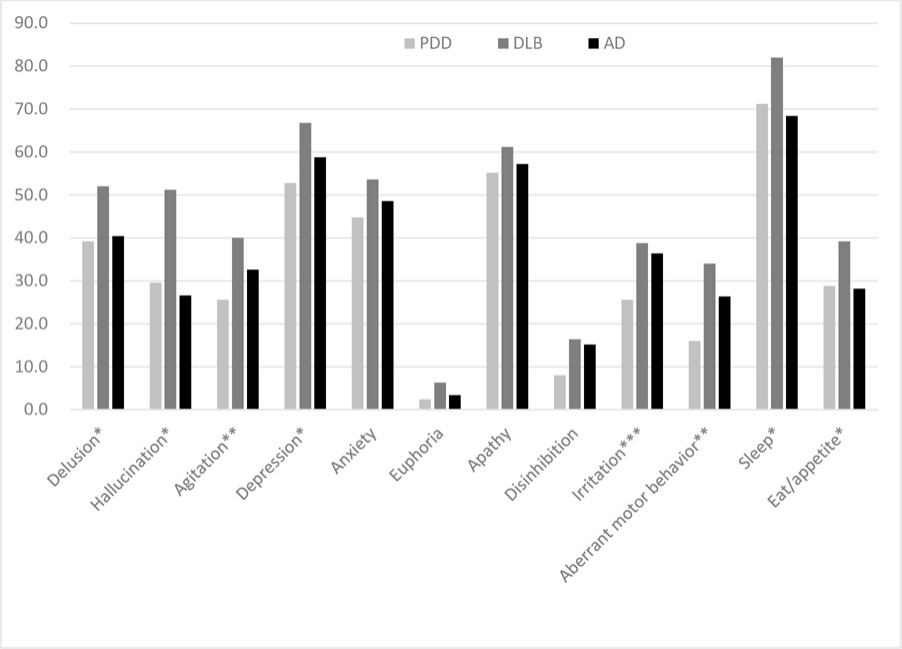
Comparison of the frequency of each item in the NPI among the PDD, DLB and AD groups adjusted for gender and use of antipsychotics. * PDD = AD < DLB; ** PDD < AD < DLB; *** PDD < AD = DLB.

Fig 1 demonstrates the frequencies of neuropsychiatric symptoms among the three groups. In the PDD group, sleep disorders were the most common symptom (71.2%), followed by apathy (55.2%) and depression (52.8%). In the AD group, similar to the PDD group, sleep disorders (68.4%), apathy (57.2%) and depression (58.8%) were the three most common symptoms. In the patients with DLB, sleep disorders were the most common symptom (82.0%), followed by depression (66.8%) and apathy (61.6%). Comparisons of the 12 items in the NPI revealed that the PDD group had lower rates of delusions, hallucinations, agitation, depression, anxiety, irritation, aberrant motor behavior, sleep, and eat/appetite than the DLB group. The PDD group had a similar neuropsychiatric profile to the AD group, with only a lower frequency of irritation compared to the AD group.

Comparisons of the severity of each item in the NPI revealed that the PDD group had lower severity of delusions (*p* = 0.004), hallucinations (*p* < 0.001), agitation (p = 0.015), and euphoria (p = 0.040) than the DLB group. The PDD group had a similar severity of neuropsychiatric symptoms to the AD group, with only a lower severity score of euphoria (p = 0.025) compared to the AD group.

Table 3 summarizes the results of multiple logistic regression analysis for patients with more severe neuropsychiatric symptoms (defined as NPI ≥ 21, N = 440, 50.3%) compared to those with milder symptoms (defined as NPI ≤ 20, N = 435, 49.7%). The results revealed that younger age, more advanced stage, and a diagnosis of DLB were associated with severe neurological symptoms of dementia.

**Table 3:** Multivariate risk estimates (ORs) for all patients with NPI ≥ 21 (N = 440, 50.3%) compared to those with NPI ≤ 20 (N = 435, 49.7%).

Characteristics		No (%)	OR (95% CI)	*p*
Diagnosis	AD	500 (57.1)	1.20 (0.78–1.84)	NS
	DLB	250 (28.6)	**2.62 (1.63–4.21)**	**< 0.001**
	PDD	125 (14.3)	1	
CDR	0.5	172 (19.7)	**0.18 (0.10–0.32)**	**< 0.001**
	1	362 (41.3)	**0.43 (0.29–0.65)**	**< 0.001**
	2/3	341 (39.0)	1	
Gender	Female	513 (58.6)	1.36 (0.99–1.88)	NS
	Male	362 (41.4)	1	
Age, year	≤ 78	440 (50.3)	**1.38 (1.02–1.88)**	NS
	> 78	435 (49.7)	1	
Education, year	≤ 5	381 (43.5)	0.81 (0.59–1.11)	**< 0.001**
	> 5	494 (56.5)	1	
IADL	≤ 3	398 (45.5)	1.20 (0.82–1.75)	NS
	> 3	477 (54.5)	1	
MMSE	≤ 15	432 (49.4)	1.27 (0.90–1.81)	NS
	> 15	443 (50.6)	1	

OR: Odds ratio; CI: Confidence interval; NS: Non-significance; AD: Alzheimer’s disease; DLB: Dementia with Lewy bodies; PDD: Parkinson’s disease dementia; CDR: Clinical Dementia Rating Scale; IADL: Instrumental Activities of Daily Living; MMSE: Mini-Mental State Examination.

## Discussion

In the current study, we used a case-control design matched with age and disease severity and recruited a relatively large sample of patients (N = 875) to compare the neuropsychiatric symptoms of PDD with those in DLB and AD. Our findings revealed a similar presentation of neuropsychiatric symptoms in PDD compared to AD but not DLB. As the demographic data among the three groups were significantly different, we further adjusted for gender and the current use of antipsychotics, and both the PDD and AD groups still presented with milder symptoms in several items of the NPI compared to DLB. These findings may provide evidence that patients with PDD have less frequent and less severe neuropsychiatric symptoms than patients with DLB, but that this may not necessarily be reflected in disparity in the overall severity of dementia as concluded by the MDS task force group [3].

Relatively few studies have directly compared the manifestation of neuropsychiatric symptoms of PD/PDD with other types of dementia, and these studies have included relatively small sample sizes. Starkstein et al. (1996) compared psychiatric differences between PDD (N = 33) and AD matched with age, gender, and MMSE score, and found a higher rate of depression and lower rate of mania in patients with PDD than in those with AD. In their study, psychotic symptoms were not significantly different between these two groups [11]. Kao et al. (2009) compared cognitive and neuropsychiatric symptoms using the 12-item version of the NPI among patients with PD (N = 14), multiple system atrophy (N = 12) and DLB (N = 14), and found that patients with DLB had the most frequent neuropsychiatric symptoms on the NPI with the exception of higher frequencies of anxiety and sleep disturbance in patients with PD [12]. Aarsland et al. (2001) compared neuropsychiatric symptoms among patients with AD (N = 42) and PDD (N = 42) using the 10-item version of the NPI, and found that the PDD patients had more severe hallucinations whereas the patients with AD had more severe aberrant motor behavior, agitation, disinhibition, irritability, euphoria, and apathy [13]. Consistent findings of a higher frequency of hallucinations in patients with DLB/PDD compare to patients with AD was also reported by Noe et al. in 2004 [14]. Our results demonstrated similar findings but with some differences that compared with AD, a significantly higher frequency of hallucinations was found in patients with DLB but not with PDD (see Fig 1). Aarsland et al. (2001) also compared psychotic symptoms among patients with PD (N = 83), PDD (N = 48) and DLB (N = 98), and found significantly higher frequencies of both delusions and hallucinations in the patients with DLB compared to those with PDD [15]. Our results revealed similar findings that frequent and less severe neuropsychiatric symptoms were found in the patients with PDD compared to those with DLB in almost all of the items in the NPI except for euphoria, apathy, and disinhibition.

With a relatively large sample size but without matching age or disease severity, López-Pousa et al. (2007) conducted a study in Spain and reported higher frequencies of delusions and hallucinations in patients with DLB compared to those with PDD or AD [16]. Another study conducted by Johnson et al. (2011) compared the neuropsychiatric symptoms among PDD, DLB, AD, VaD, and mixed type dementia revealed that PDD had the lowest levels of neuropsychiatric symptoms [17]. A most recent study conducted by Hashimoto et al. (2015) enrolled a relative large number of patients from multiple centers in Japan revealed that overall neuropsychiatric symptoms were more severe in DLB compared to AD. They also reported no significant difference in severity of neuropsychiatric symptoms according to NPI across CDR staging in the DLB group. However, the severity of neuropsychiatric symptoms is parallel with dementia stage in the AD group [19]. These large sample studies, together with our study, investigated the presentation of neuropsychiatric symptoms among the three types of dementia and the conclusion is still controversial. The only consistency is that patients with DLB have more severe neuropsychiatric symptoms; however, it deserves attention that more severe neuropsychiatric symptoms in DLB may be resulted partly from the occurrence of visual hallucinations and/or REM sleep behavior disorder, which are part of the diagnostic criteria for DLB and have expected to be higher in the DLB group.

Complex interaction of the pathologies of Alzheimer’s disease (amyloid-β plaques and tau-containing neurofibrillary tangles) and Lewy body disease (α-synuclein) are enthusiastically discussed in recent studies [24, 25]. Evidence on the concurrence of Alzheimer’s pathologies or Alzheimer type atrophy in PDD is robust [25–27] and the Alzheimer’s pathologies may act synergistically with Lewy body-related pathology to confer a worse prognosis [27]. A study also suggested that Alzheimer’s pathologies may contribute to the progression to dementia in PD patients, especially in the late onset PD group [26]. Severity of neuropsychiatric symptoms in PD may parallel with the severity of cognitive dysfunction [28], in other words, neuropsychiatric symptoms in PD could possibly be influenced by the Alzheimer’s pathology. In the current study, most of our PDD patients were late onset and thus might have higher probability of the concomitant of Alzheimer’s pathology. In contrast to PDD, the clinical manifestation of DLB might not be necessarily influenced by Alzheimer’s pathologies [29, 30], on the contrary, one study demonstrated less severe visual hallucinations in low likelihood DLB group (high Alzheimer’s pathology group) than in high likelihood one [30]. Differences in pathology between PDD and DLB are another possible explanation for our finding. In this view, PDD and DLB might be regarded as different disease entities.

Finally, we analyzed the association factors of severe neuropsychiatric symptoms among all dementia patients and our results revealed that severe neuropsychiatric symptoms were associated with younger age, more advanced stage, and a diagnosis of DLB. The finding that more severe neuropsychiatric symptoms were associated with more advanced stage of dementia is compatible with findings from most of the previous studies. However, the association with younger age is not according with previous studies [31–33]. The patients with DLB in our study presented with more severe and more frequent neuropsychiatric symptoms and also demonstrated a higher caregiver burden score. This suggest that caregivers of dementia patients with severe neuropsychiatric symptoms have higher stress and burden, which is consistent with several other studies on the association of the severity of neuropsychiatric symptoms and caregiver distress/burden [34–36].

There are several limitations to this study. First, the mean age of our PDD patients was relatively old (95.2% > 65 years old), and nearly half (46.4%) of the PDD patients had a short duration of parkinsonism (2–3 years). The pathological and clinical presentation of early- or late-onset PD patients may be different [26]. Therefore, there may have been selection bias in the patients with PDD, and our findings may not be generalizable to all patients with PDD. Second, two cognitive screening tests (MMSE and CASI) were used for the determination of cognitive dysfunction and more detailed cognitive profiles of the patients are lacking. Both MMSE and CASI do not allow to define level II diagnosis criteria for Parkinson's disease dementia according to the MDS clinical criteria. Therefore, there may have been lower accuracy for the confirmation of dementia in Parkinson’s disease. Third, the comparison of associated factors between patients with severe and mild neuropsychiatric symptoms in our study was cross-sectional. Therefore, a causal relationship between factors and dementia could not be investigated.

In conclusion, the results from this study provide evidence that the neuropsychiatric symptoms in PDD are more like those in AD compared to DLB, and that this may not necessarily be reflected in disparity in the overall dementia severity. Severe neuropsychiatric symptoms in these degenerative types of dementia were associated with a younger age, more advanced stage of dementia, and a diagnosis of DLB.
